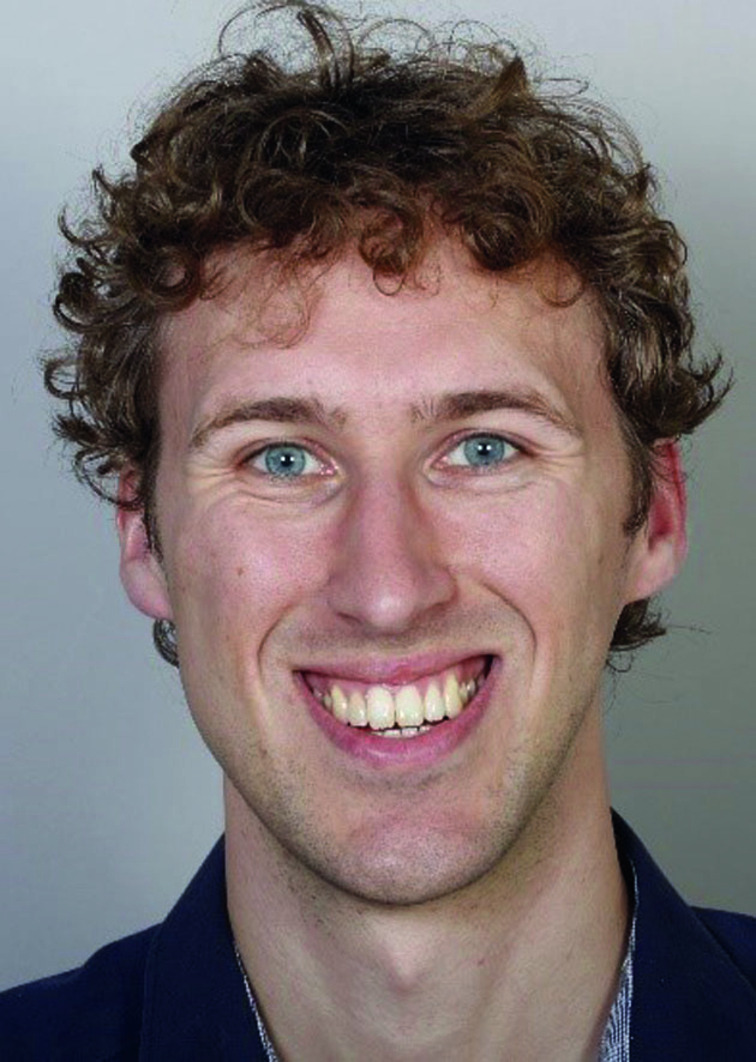# Sustainable Hydrometallurgical LFP Battery Recycling: Electrochemical Approaches

**DOI:** 10.1002/cssc.202402699

**Published:** 2025-03-19

**Authors:** Cody B. Van Beek, Eda Yilmaz, Devin H. A. Boom

**Affiliations:** ^1^ Environmental Modelling Sensing & Analysis (EMSA) Netherlands Organization for Applied Scientific Research (TNO) Princetonlaan 6 3584 CB Utrecht, The Netherlands

**Keywords:** LFP battery recycling, electrohydrometallurgy, redox mediator, lithium, sustainable processes

## Abstract

Lithium‐ion batteries (LIBs) are crucial for the energy transition, particularly with the rising demand for electric vehicles. Among different battery technologies, lithium iron phosphate (LFP) batteries have been attracting considerable attention in recent years due to their safe chemistry and relatively cheaper and abundant material composition. As LFP manufacturing is set to increase significantly, a proper end‐of‐life treatment of these batteries becomes essential to achieve circularity and minimize environmental impacts. However, recycling of LFP batteries is economically challenging because they do not contain many valuable transition metals. This Concept article focuses on recycling of LFP batteries, and explores whether economically viable LFP recycling can be made possible via improvement of recycling processes. Currently, hydrometallurgical recycling processes with inexpensive oxidants for leaching valuable lithium show potential, compared to pyrometallurgical processes. However, these processes still consume large amounts of chemicals. Electrochemical recycling methods that do not require continuous addition of external reagents, or minimize waste production, could lead to more sustainable and economically viable solutions for LFP battery recycling. In addition, combining these processes with other sustainable electrochemical technologies such as green hydrogen production, brine desalination and chemical production is a promising strategy to increase overall energy and product efficiency.

## Graphical abstract

Lithium iron phosphate (LFP) batteries are gaining attention for their safety and cost‐effectiveness. However, recycling them is challenging due to low intrinsic value of the materials. The utilization of electrochemical methods in hydrometallurgical processes show potential for sustainable recycling. Combining these with technologies like green hydrogen production could enhance efficiency and economic viability.

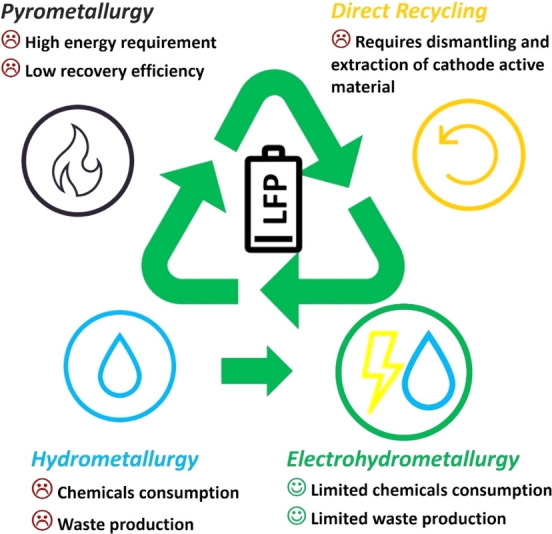

## Introduction

Lithium‐ion batteries have become an indispensable part of the energy transition, especially as a consequence of an increasing demand for electric vehicles. Lithium‐ion (Li‐ion) battery manufacturing is expected to increase considerably in the next decade and this will cause a domino effect in the whole value chain.[^1^, ^2^] After these batteries reach their end of life, they can be processed through different R‐strategies, such as refurbishment. Finally, recycling will be the last step to recover the materials and to keep them in the circular economy. While recycling of Li‐ion batteries (LIB) is an indispensable step, the volatility of metal prices, energy intensive processes, and low recovery efficiencies pose a challenge to make a profitable business case in this sector. Profits for recycling activities depend very much on the economic value of the materials recovered from the waste batteries and the operation costs of the recycling processes.^[3]^ Battery chemistries such as LCO (LiCoO_2_), NMC (Li(Ni_x_Mn_y_Co_z_)O_2_) and NCA (Li(Ni_x_Co_y_Al_z_)O_2_) present a good opportunity for profitable recycling operations, since they contain valuable metals such as lithium, cobalt and nickel. On the other hand, the newly emerging LFP (LiFePO_4_) batteries lack economic incentive for recycling because they do not contain a lot of valuable transition metals.

The market share of the LFP batteries is expected to grow substantially in the coming years, due to their advantages over existing battery chemistries such as higher safety, low price and good stability.[^2^, ^4^] In addition, these batteries can help accelerate the energy transition and thus minimize adverse effects of climate change. However, if the waste streams that will follow are not addressed properly, they can cause other environmental concerns in the future. Therefore, the development of both sustainable and economically viable recycling processes for LFP batteries are essential to maximize their contribution to the energy transition. In recent years, the number of reports addressing this challenge has been growing, with varying approaches presented by both academia and industry.

Considering the cost and energy requirement of recycling steps, the most viable option for LFP recycling is proposed to be direct recycling.^[5]^ Direct recycling involves physical extraction of cathode and anode active materials separately from the end‐of‐life battery cells, while keeping the materials (e. g. cathode crystal structure) intact as much as possible. This is followed by regeneration of the materials with minimal processing into ready to use battery active materials.[^6^, ^7^, ^8^] However, efficient separation of cathode active materials from the rest of the cell components is crucial for direct recycling processes, which poses a big challenge to achieve in large scale with the current battery cell structures. On the other hand, hydrometallurgical processes can be more flexible and can recover battery materials from shredded battery cells. In order to decrease the environmental impact and to increase the economic viability of these hydrometallurgical processes, development of new techniques is essential. This Concept article will present a brief overview on the different strategies for hydrometallurgical LFP battery recycling, and will pay special attention to electrochemical approaches as an opportunity to simultaneously lower the environmental impact of the recycling process and overall process costs.^[9]^

## Current Practices for LFP Recycling

The two most explored processes for LIB recycling are pyrometallurgy and hydrometallurgy, or a combination thereof. Other approaches include direct recycling, and less widely studied solvometallurgy and biometallurgy.[^5^, ^10^, ^11^] When comparing energy and materials consumption of these different recycling strategies, direct recycling appears as the most efficient process.^[5]^ This is involves regeneration of the materials with minimal processing into ready to use battery active materials.[^6^, ^7^, ^8^] This recycling strategy is particularly attractive for LFP batteries, since it can decrease the recycling costs significantly, and potentially make LFP battery recycling economically more feasible.[^12^, ^13^] However, the upscaling of direct recycling processes faces technical challenges. A direct recycling process typically requires manual separation of the cathode and anode active materials from the rest of the cell components. During laboratory scale experiments manual extraction of the cathode and anode materials is possible, but on industrial scale, to the best of our knowledge, currently there is no advanced automated system that can extract these materials. Although future developments, e.g. batteries designed for recycling according to safe‐and‐sustainable‐by‐design principles,^[14]^ could enable direct recycling on industrial scale,^[15]^ direct recycling does not seem feasible for addressing current waste streams.

Compared to direct recycling, pyrometallurgical recycling processes have much higher emission and energy consumption.^[5]^ However, these pyrometallurgical processes are readily used in the industry since these are mature technologicallies and can rely on already existing installations. Even though pyrometallurgy is a seemingly easy to implement technology for LFP battery recycling, the value created via this route is very limited: lithium is typically lost in the slag, and copper, present as anodic current collector, is the only remaining valuable metal of the battery that can be extracted.[^16^, ^17^, ^18^, ^19^, ^20^] Even with emerging technologies like lithium volatilization, pyrometallurgical LFP recycling remains economically and environmentally challenging.^[21]^

On the other hand, hydrometallurgical recycling is a more viable option for LFP batteries because one can recover not only copper, but also lithium and other metal salts via this process. In practice, hydrometallurgical processes can extract multiple materials from black mass (the black powder containing cathode and anode active materials obtained by shredding the batteries and physical separation steps). Unlike direct recycling processes, hydrometallurgy can be more easily scaled up for larger operations. Hydrometallurgy is a robust technique that can be adapted to varying input compositions. Often, the recovered lithium and iron containing salts can be directly used for production of new LFP cathode active materials.

A typical hydrometallurgical process involves an acid leaching step in the presence of an oxidant, to leach Li from the LFP cathode material leaving behind insoluble FePO_4_. For such processes, inorganic acids like H_2_SO_4_,^[22]^ H_3_PO_4_,^[23]^ HCl,^[24]^ or organic acids like oxalic acid,^[25]^ citric acid,[^26^, ^27^] acetic acid,^[28]^ and formic acid[^29^, ^30^] can be used as acid, combined with H_2_O_2_ as the most commonly used oxidant. H_2_O_2_ can also be used as an oxidant without acid.^[31]^ Various comprehensive summaries describing different leaching conditions for hydrometallurgical processes have already been published as review papers.[^4^, ^12^, ^13^, ^32^, ^33^] While the concentrations and the reaction conditions can vary slightly when different types of acids are used, the general trend is that the acid and oxidant are used in excess to ensure high lithium recovery efficiency.^[34]^ In addition, typically the leaching process is performed at elevated temperatures and thus hydrometallurgical processes can also have a high energy consumption.[^28^, ^35^, ^36^, ^37^, ^38^, ^39^] The abovementioned typical reaction conditions can lead to unfavorable consequences such as; increase in CAPEX due to a requirement for specialized equipment, and an increased environmental impact because of the intense chemical use and the production of more waste.[^5^, ^19^] In order to make LFP recycling as economically viable as possible, these consequences need to be avoided. One possible solution toward this is the development of electrochemical hydrometallurgical processes of which several recent advancements are described in the sections below.

## Alternative Oxidative Lithium Leaching Methods

Using only stoichiometric amounts of oxidant and acid during the leaching process can potentially increase the sustainability and economic viability of LFP recycling. However, the unstable nature of H_2_O_2_ is a big challenge when using stoichiometric amounts, and thus for achieving a high recovery efficiency for lithium. Nevertheless, selective leaching of lithium from LFP cathode material is not only limited to the use of H_2_O_2_ as oxidant in combination with an (in)organic acid. Other chemical oxidants, with and without acids, have been explored for the oxidation of LFP with selective leaching of lithium ions, which will be discussed in the following section.

**Figure 1:**
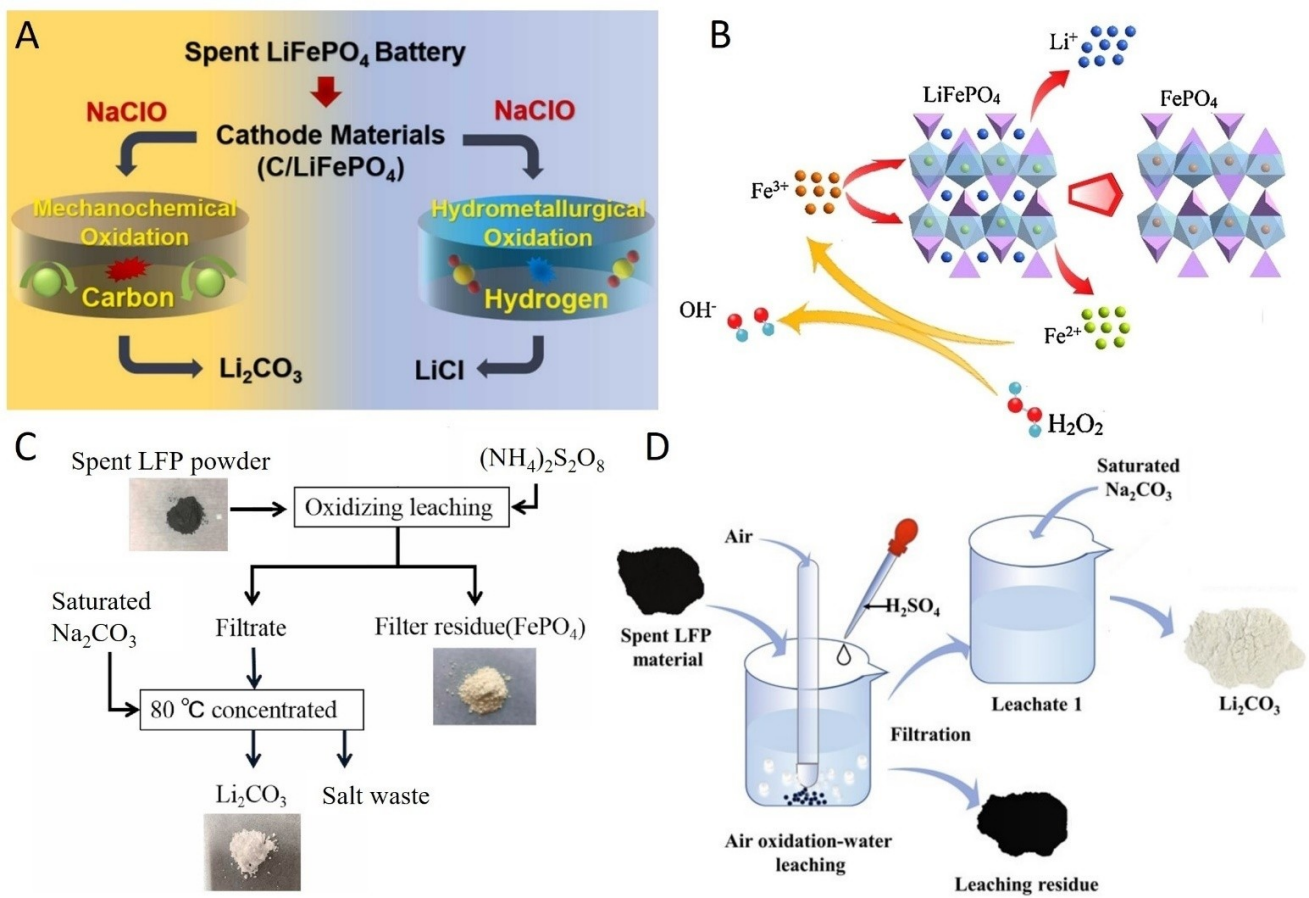
Various methods that utilize the chemical oxidation of LFP for selective lithium leaching using air as an oxidant (A), NaClO in a wet chemical or mechanochemical oxidation process (B) or ball‐milling process with (NH_4_)_2_S_2_O_8_ as oxidant (C) or Fe^3+^ ions with or without H_2_O_2_ (D). Subfigure A was reprinted from ref. [40], subfigure B was adapted for clarity from ref. [42], subfigure C was adapted for clarity from ref. [44], and subfigure D was adapted for clarity from ref. [46], all with permission from Elsevier.

As an alternative to the commonly used hydrogen peroxide, Liu and co‐workers studied the well‐known oxidant NaOCl for the selective dissolution of lithium by oxidation of LFP through either a mechanochemical or hydrometallurgical oxidation process, see Figure 1‐A.^[40]^ In the mechanochemical oxidation process, Li_2_CO_3_ was formed, which is proposed to proceed through initial formation of Li_2_O as an intermediate, which subsequently reacts with CO_2_ that is formed by oxidation of graphite present in the spent LFP (sLFP) cathode material. During the hydrometallurgical process, NaClO and LFP cathode material are mixed, resulting in LiCl as lithium containing product. A similar hydrometallurgical process based on HCl/NaOCl leaching was reported by Liu and co‐workers, which showed promising results for selective lithium leaching and isolation of Li_2_CO_3_ as product.^[41]^

Due to its cost‐attractiveness, inexpensive Fe^3+^ salts have also been investigated as oxidant. Dai and co‐workers proposed two methods that utilize Fe_2_(SO_4_)_3,_ an Fe^3+^ source, in a stoichiometric process or with a catalytic amount of Fe^3+^ in combination with the oxidizing H_2_O_2_, see Figure 1‐B.^[42]^ The addition of a stoichiometric amount of Fe_2_(SO_4_)_3_ to LFP cathode material resulted in above 97 % of the lithium ions in solution after 30 min. After removal of the iron ions, lithium was recovered as Li_2_CO_3_. Jiang and co‐workers also utilized Fe^3+^ to oxidize LFP in a process in which LFP and lithium cobalt oxide (LCO) cathode active materials were mixed in a 0.5 M H_2_SO_4_ solution. Under these conditions, LFP partially dissolves, and the dissolved Fe^2+^ ions reduce the Co^3+^ in LCO, resulting in dissolved Fe^3+^, Li^+^ ions and Co^2+^ ions, respectively. In this stage, the *in situ* formed Fe^3+^ ions act as a catalyst by oxidizing LFP as Fe^3+^ ions and reducing LCO as Fe^2+^ species, which leads to dissolution of lithium, iron and cobalt ions in this acidic solution. It is claimed that this process reduces the amount of chemicals (oxidants and acid) required and therefore has a reduced environmental footprint.

Liu and co‐workers describe a mechanochemical oxidation process of LFP that uses Na_2_S_2_O_8_ as oxidant and does not require the use of acid.^[43]^ This strong oxidant rapidly oxidizes LFP and selectively forms Li_2_SO_4_ in 5 min, which subsequently can be dissolved in water. Afterwards, lithium is recovered by precipitation as Li_3_PO_4._ Similarly, Chen and co‐workers disclosed a hydrometallurgical LFP recycling process that utilizes (NH_4_)_2_S_2_O_8_ as oxidant for 30 min.^[44]^ After filtration, lithium is recovered as Li_2_CO_3_, see Figure 1‐C. Shentu and co‐workers also describe the use of (NH_4_)_2_S_2_O_8_ as oxidant in aqueous solution, leading to selective formation of Li_2_SO_4_ which subsequently can be converted to LiOH and H_2_SO_4_ by an electrochemical process.^[45]^

Another approach to increase the sustainability of a chemical process, is to use abundant and readily available reagents. An attractive and abundant oxidant that has been studied is using oxygen from air by Jin and co‐workers. Thermodynamic analysis showed that oxygen is able to oxidize LFP with selective release of lithium ions under mildly acidic conditions with formation of FePO_4_ residue, see Figure 1‐D. The influence of several parameters on the efficiency of the process was investigated under atmospheric air pressure,^[46]^ resulting in more than 99 % leaching of lithium in 5 h and under 0.4 MPa of air pressure resulting in 99 % leaching of lithium in 5 h.^[47]^ Zhu and co‐workers reported on a similar air oxidation method but they use small amounts of H_3_PO_4_,^[48]^ whereas Jin and co‐workers use H_2_SO_4_.

## Electrochemical Methods

### Mediated Electrochemical Leaching

**Figure 2:**
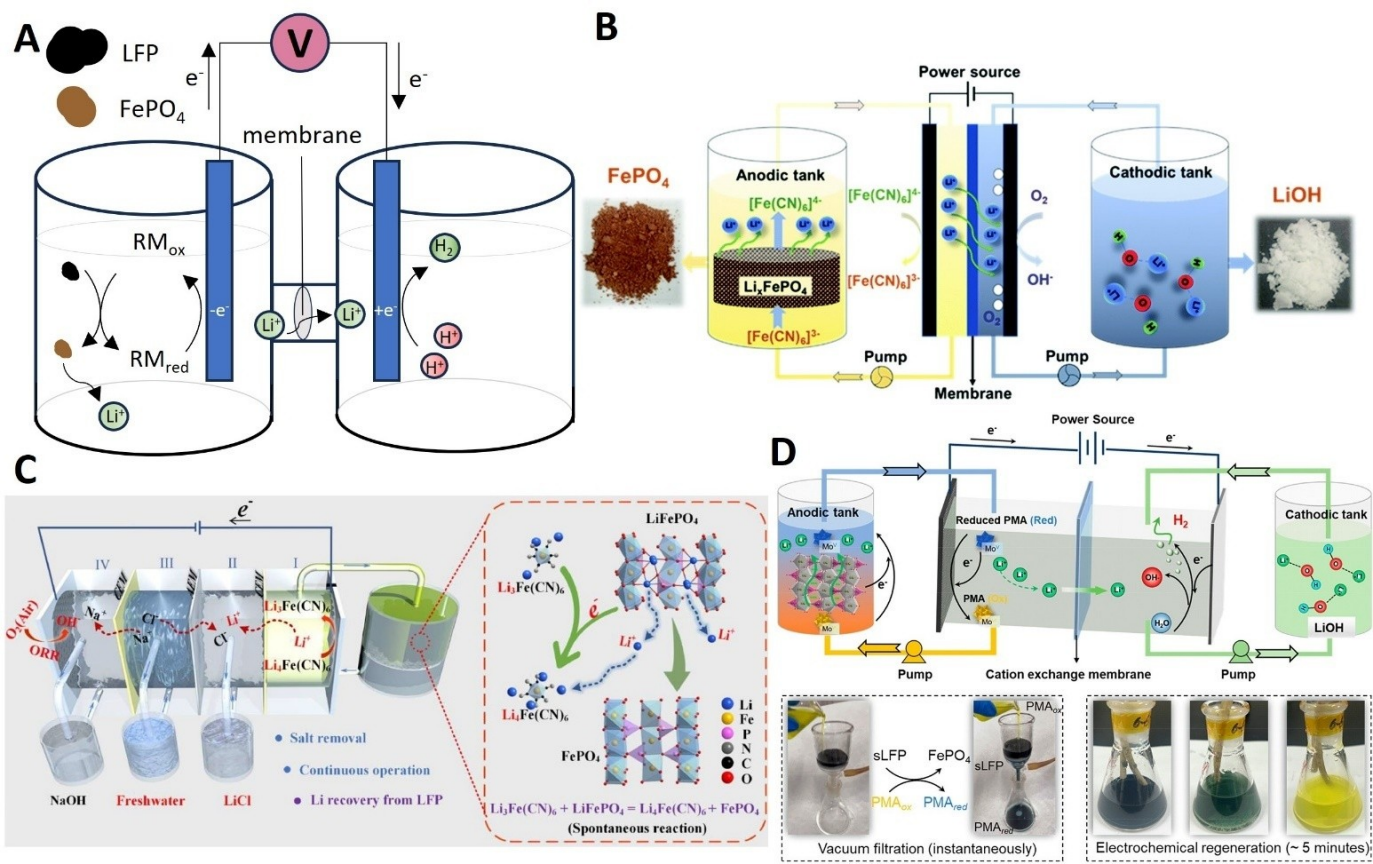
Schematic representation of a membrane separated cell for indirect electrochemical oxidation of LFP using a redox mediator (A) and various examples of LFP oxidations using RMs. A method with a cation exchange separated cell using [Fe(CN)_6_]^3−^ as RM (B) and a method describing redox flow desalination using Li_3_[Fe(CN)_6_] as RM (C) and a method with a cation exchange membrane separated cell mediated by a polyoxometalate (PMA) (D). Subfigure B is reprinted from ref. [51] with permission of RSC, subfigure C is reprinted in part from ref. [35], subfigure D is reprinted in part from ref. [55].

The state of the art hydrometallurgical recycling processes for LFP, as well as the alternative oxidative leaching methods described above, require stoichiometric or excess oxidant and acid/base. This gives the opportunity for development of more sustainable and more economically viable recycling processes. In view of that, mediated electrochemical oxidation processes have been investigated recently, which utilize electrons instead of stoichiometric/excess reagents. Such processes require the use of redox mediators (RMs), which can be dissolved molecules, ions or complexes, that act as electron shuttles. A suitable RM is electrochemically activated by oxidation at the anode and then oxidizes the sLFP powder. Because RMs display reversible electrochemical properties and are generally stable, only a catalytic amount is required (see Figure 2‐A). Various counter reactions can take place at the cathode, but typically proton reduction takes place, producing hydrogen gas. Notably, the concept of using a RM to reversibly oxidize/reduce LFP/FePO_4_ to be able to obtain high energy density batteries with LFP using osmium‐based RMs was already established two decades ago.^[38]^

Recently, several RMs have been investigated for their role in the indirect electrochemical oxidation of sLFP. The most studied RM for indirect electroleaching of LFP is the ferricyanide/ferrocyanide couple ([Fe(CN)_6_]^3−^/[Fe(CN)]_6_^4−^). This RM can be used in neutral or basic solution, but is unstable in acidic medium.[^49^, ^50^] This allows for the opportunity to selectively leach lithium ions from LFP and leave insoluble FePO_4_ undissolved under these conditions. Yu and co‐workers reported on using a solution of [Fe(CN)_6_]^3−^ as RM in a two‐compartment electrochemical cell that uses carbon felt electrodes. The electrodes are separated by a Nafion 117 cation exchange membrane (CEM), which lets cations (Li^+^) freely diffuse towards the cathodic compartment.^[51]^ A 0.2 M Li_3_[Fe(CN)_6_] electrolyte was used for the anodic tank and a 0.1 M LiOH electrolyte was used for the cathodic tank, which was bubbled with oxygen, see Figure 2‐B. The oxidation of [Fe(CN)_6_]^4−^ is accompanied with the oxygen reduction reaction (ORR) at the cathode, resulting in the formation of hydroxide ions in the cathodic compartment. Diffusion of lithium ions through the membrane during electrolysis results in the formation of LiOH as product in the cathodic compartment. Another contribution from the same group describes combining the LFP recycling process with proton reduction on the cathode, to be able to couple a useful oxidation reaction to hydrogen production in an electrolyser.^[52]^ This way, hydrogen gas can be produced at large scale and a lower amount of electricity is needed compared to conventional water electrolysis due to a lower cell potential. This was performed in a two compartment flow cell using a carbon cloth anode with 0.5 M Li_2_SO_4_ and 0.1 M Li_3_[Fe(CN)_6_] as anolyte, and Pt/C cathode with 0.1 M LiOH catholyte. A charge‐reinforced ion selective (CRIS) membrane was used as separator, which prevents diffusion of the RM through the membrane. The cell could be operated at 300 mA cm^−2^ at 1.53 V resulting in a 43 % reduced energy consumption to produce hydrogen gas compared to conventional water electrolysis, and is also more profitable due to additional LiOH production.

Another approach to make LFP recycling more economically viable is by incorporating multiple electrochemical processes into one operation unit. Shan and co‐workers describe a RM approach to recycle LFP, and couple it with a redox flow desalination system.^[35]^ Here, a brine solution (10.000 ppm NaCl) is desalinated simultaneously with LFP oxidation to obtain fresh water, LiCl and NaOH solutions, and FePO_4_ as products. The complex set‐up utilized for this process consists of a four‐compartment electrochemical cell separated with CEMs (between compartments I–II and III–IV) and an AEM (between compartments II–III) (see Figure 2‐C). The cathode consists of a carbon cloth that is coated with a Ir/C layer as an efficient catalyst for the ORR and at the other side plain carbon cloth is used as anode. During operation of the redox flow cell, oxygen is reduced at the cathode and [Fe(CN)_6_]^4−^ is oxidized at the anode which can subsequently react with LFP. Lithium was recovered as LiCl solution with 70 % efficiency in this small scale setup.

Li and co‐workers also worked on an innovative redox flow‐based system that is able to combine lithium extraction from LFP with lithium extraction from brine/sea water in two different cycles.^[53]^ Li_3_[Fe(CN)_6_] was employed as RM (0.5 M), which was used in one compartment of a three compartment cell. The compartments are separated by a modified Nafion‐117 CEM with high selectivity towards lithium ions and an AEM for chloride ions transport. The middle chamber is connected to either a lithium brine/sea water tank or a lithium recovery solution tank. The third chamber uses the ZnCl_2_/Zn couple connected to a ZnCl_2_ solution tank (0.25 M + 1 M LiCl). During the discharge phase of the redox flow system, electrical energy is released by oxidation of Zn and reduction of Li_3_[Fe(CN)_6_] while lithium ions are captured by the RM forming Li_4_[Fe(CN)_6_] in solution. During the charge phase, Li_4_[Fe(CN)_6_] is oxidized at the anode and lithium is released in the lithium recovery solution by diffusion through the modified CEM, concomitant with metallic zinc deposition at the cathode. The oxidized Li_3_[Fe(CN)_6_] is then pumped to a LFP containing tank and spontaneously reacts with LFP, releasing lithium ions and forming FePO_4_. The charge/discharge phases allow to use the system as a redox flow battery coupled to lithium recovery from a solid state and solution simultaneously.

Other RMs have also been described in the literature. An innovative self‐powered device was constructed by Zhang and co‐workers that uses the ClO^−^/Cl^−^ couple as RM for LFP oxidation.^[54]^ Using battery components, a triboelectric nanogenerator (TENG) was built, which utilizes wind energy to generate electricity. The produced electricity was applied to electrochemically produce ClO^−^ from a 0.6 M NaCl electrolyte solution. The electrochemical cell described contains graphite as anode material and platinum metal as cathode material for proton reduction in a two compartment cell separated by a CEM. ClO^−^ can oxidize LFP at pH 6 and lithium was recovered in a subsequent step by precipitation as Li_2_CO_3_, constituting an alternative way to obtain high purity Li_2_CO_3_.

Zhu and co‐workers described another RM, phosphomolybdic acid (PMA), a member of the polyoxometalates, which is effective in oxidizing LFP (see Figure 2‐D).^[55]^ A two compartment flow cell consisting of a carbon felt anode, a Nafion 117 CEM and a ruthenium‐plated titanium plate as cathode was used. 10 mM PMA solution at pH 2 was used as anodic electrolyte and 0.1 M LiOH as cathodic electrolyte. The oxidized and reduced forms of PMA have a distinct yellow and blue color, respectively. After rapid reaction of the yellow colored PMA with LFP, an intense blue solution is formed and the process can be monitored visually. A pH of 2 or lower is required to keep the redox‐active Keggin structure of PMA stable, resulting in some dissolution of iron and phosphate. LiOH is obtained in the cathodic chamber as product via diffusion of lithium through the CEM and formation of hydroxide at the cathode. The process enables LFP recycling, LiOH formation and hydrogen gas formation with a low‐energy consumption.

While many of the described mediated electrochemical leaching methods above seem promising as more environmental friendly options for LFP leaching, possible limitations should also be addressed before large scale use is possible. RMs could suffer from poor stability under the reaction conditions and/or be adsorbed on graphite/particles present in black mass. Most of the literature examples described in this Concept article use LFP/manually extracted LFP powders and the stability of RMs in reaction with black mass is not described. Black mass contains more components/impurities, which may react with the RM. In addition, to be able to reuse the RM, it should be separated from the lithium containing solution, which could be challenging.

### Direct Electrochemical Leaching

**Figure 3:**
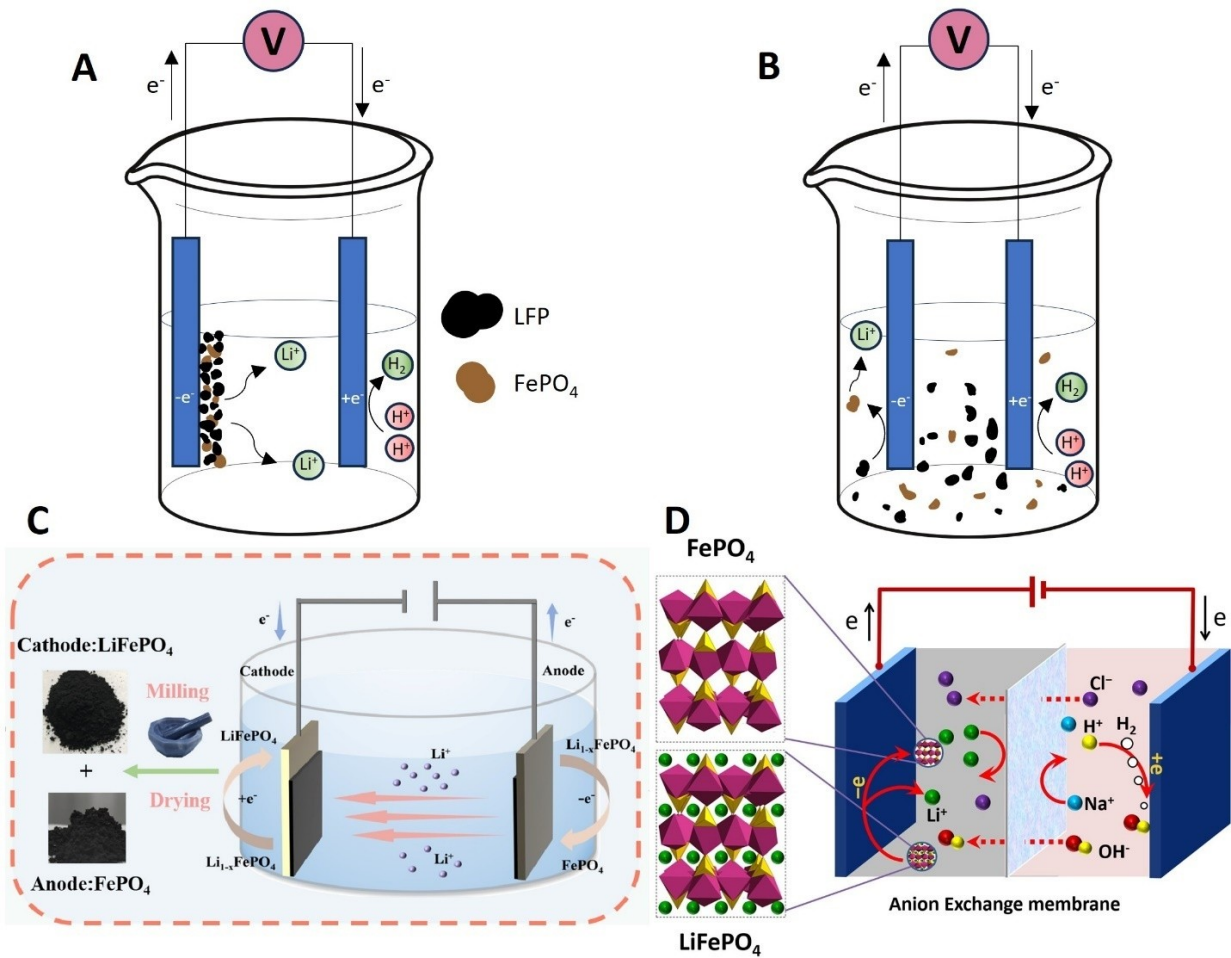
Two methods of direct electrochemical leaching of LFP with direct oxidation of LFP coated on an anode (A) or oxidation of LFP particles by slurry electrolysis (B) and two examples describing direct electrochemical de‐lithiation/re‐lithiation of LFP coated on both electrodes (C) or anion exchange membrane separated slurry electrolysis of LFP (D). Subfigure C is reprinted in part from ref. [59] and subfigure D is reprinted from ref. [61], both with permission from Elsevier.

In contrast to indirect electrochemical methods to oxidize LFP, also direct electrochemical methods have been investigated that do not utilize a RM. Interestingly, this concept is closely related to the charging process of an LFP battery, during which the LFP cathode active material is oxidized resulting in de‐intercalation of lithium ions and FePO_4_ is left on the cathode current collector. In such an approach, sLFP powders can be coated on an electrode and these prepared electrodes are subsequently used as anodes in an electrochemical cell. Through a direct electrochemical oxidation of the coated sLFP powder, lithium ions are released, see Figure 3‐A. The remaining electrode material consists out of a FePO_4_ coating, which does not dissolve in mildly acidic or neutral solution. This FePO_4_ residue can potentially be collected and reused in the synthesis of new LFP powder.

Wang and coworkers reported a direct electrochemical LFP leaching method that utilizes a graphite felt electrode that is coated with sLFP powders.^[56]^ The coated anode was placed in a cell with H_3_PO_4_ as electrolyte, which leaches both lithium and iron ions from the anode, while proton reduction occurs at the cathode. In a series of precipitation steps FePO_4_, Li_3_PO_4_ and NH_4_H_2_PO_4_ were recovered as products. Lithium and iron were both leached above 96 % and Li_3_PO_4_ was recovered with more than 93 % with an electricity consumption of <600 kWh/t sLFP. This is an order of magnitude lower than the energy demand needed for recovering the lithium by precipitation at elevated temperature. A similar direct electroleaching setup was used by Zhao and coworkers. That work describes the preparation of a titanium mesh anode coated with sLFP that is used in a cell with 0.5 M Na_2_CO_3_ electrolyte and a platinum cathode for proton reduction.^[57]^ After direct electrochemical leaching for 120 min at 80 °C, FePO_4_/C on the electrode and Li_2_CO_3_ were obtained as products (98 % recovery yield of lithium). Lv and co‐workers disclosed a similar electrochemical setup consisting of a carbon cloth anode coated with LFP and a titanium mesh cathode for proton reduction.^[58]^ The cell utilized a 0.1 M NaCl electrolyte at different potentials (2–2.4 V) and using optimal conditions resulted in more than 98 % of lithium being recovered as Li_3_PO_4_ as final product.

Yang and co‐workers described a process that can recycle sLFP, while preparing new LFP by combining anodic de‐lithiation with cathodic re‐lithiation.^[59]^ They coated a graphite plate and a titanium mesh with sLFP to prepare the anode and cathode, respectively. These prepared electrodes were placed in a cell with Li_2_SO_4_ as electrolyte and during electrolysis at 1 V for approximately 2 h to fully re‐lithiate the sLFP (see Figure 3‐C). This process does not require additional acid/base and does not result in aqueous byproducts and has a low energy consumption of 32 kWh/t sLFP. The regenerated LFP showed good electrochemical properties.

Despite not classified as a hydrometallurgical process, Zhang and co‐workers developed a direct electrochemical leaching process that also explored using sLFP powder as both the cathode and anode. They report on a paired electrolysis process in a molten carbonate salt (mixed sodium and potassium) at 750 °C under inert argon atmosphere.^[60]^ During electrolysis, the LFP cathode forms iron metal as product and on the anode Fe_3_O_4_ is formed, which both do not dissolve in the molten salt. Lithium and phosphate ions are released in the molten salt, which after processing of the molten salt will form NaLi_2_PO_4_ salt, which is further converted to Na_3_PO_4_ and Li_3_PO_4_ as final products with H_3_PO_4._

Another approach for direct electrochemical leaching of sLFP powder is through the use of slurry electrolysis. A slurry suspension of sLFP powder in an electrolyte solution is stirred in the anodic compartment. Upon contact of the anode with a LFP particle, LFP is electrochemically oxidized with release of lithium ions and formation of FePO_4_/C particles (see Figure 3‐B). These methods eliminate the need to coat LFP powders onto electrodes, thereby facilitating the scalability of a recycling process.

With such an approach, Li and co‐workers reported on a two chamber electrolytic cell separated by an AEM with ruthenium‐plated titanium electrodes and a NaCl solution as electrolyte on both sides (see Figure 3‐D).^[61]^ In the anode chamber sLFP powder was suspended in a slurry. It was mechanically stirred during electrolysis for 8 h, resulting in over 98 % leaching yield of lithium under optimized conditions. Proton reduction takes place at the cathode, resulting in the formation of NaOH as product in the catholyte and mixed LiOH/LiCl in the anodic compartment. Subsequently, lithium is recovered from this mixture as Li_2_CO_3_. In a separate study, the same group demonstrated a similar slurry electrolysis setup, but in this case separated by a CEM.^[62]^ Their two compartment cell utilizes ruthenium‐plated electrodes as anodes and cathodes with Li_2_SO_4_ and LiOH as electrolytes in the anodic and cathodic compartment, respectively. The electrolysis was performed at fixed current densities releasing lithium ions from LFP and forming hydroxide ions via the hydrogen evolution reaction at the cathode. LiOH is formed as product as lithium ions are transferred to the cathode side.

Similar to the previously mentioned studies, Li and co‐workers reported a simplified slurry electrolysis method for leaching lithium from sLFP powder. The electrochemical cell used consists of only one compartment without utilizing a membrane, and is equipped with a ruthenium‐plated titanium electrode at both sides.^[63]^ During electrolysis (optimized conditions: 4.2 V, pH 1.7, 0.05 M electrolyte, temperature 70 °C, 13 g/L sLFP), LFP is oxidized to FePO_4_ and on the cathode proton reduction takes place. Additional side reactions also take place on the electrodes. At the anode water oxidation and oxidation of dissolved Fe^2+^ ions occur, while at the cathode iron deposition occurs. Leaching efficiency of lithium above 99 % is observed and after a filtration and concentration step, lithium is recovered as Li_3_PO_4_ by precipitation with a recovery yield of 88 %. Iron is obtained as FePO_4_ or electrodeposited metallic iron.

Like the mediated methods, direct electrochemical methods also face some challenges for their practical applications. Similar to direct recycling, it is challenging to extract cathodes separately from spent LFP batteries to use for direct electrochemical oxidation. In addition, slurry electrolysis can suffer from poor selectivity in the presence of other impurities in black mass, which contain more components than manually separated LFP powders, low currents/high energy use, degradation of electrodes and low leaching efficiency at large scale.

## Economic and Environmental Considerations for LFP Recycling

In recent years studies focusing on the socioeconomic impact of batteries from manufacturing to end‐of‐life, is gaining traction.^[64]^ Such an understanding is also important for LFP batteries especially to assess the economic feasibility of their recycling. To this end, studies involving recycling often contain a techno‐economic analysis (TEA) or life cycle assessment (LCA) to evaluate this.

Current pyrometallurgical and hydrometallurgical processes for LFP battery recycling have a large environmental impact due to high energy and or chemical consumption combined with large emissions and waste production. In addition, the high costs for recycling and low economical value of the cathode active material makes it challenging to develop cost‐effective recycling methods. The studies mentioned above describing alternative chemical lithium leaching methods are directed to come up with more cost‐effective and sustainable (hydrometallurgical) processes that no longer require excess acids or oxidants. Some of these studies have also performed and reported a TEA or LCA of their process. Jin and co‐workers showed that the air oxidation process is more profitable than conventional and other LFP oxidation processes according to a preliminary economic analysis.[^46^, ^47^] In addition, it is also claimed to be more sustainable, since less reagents are required for the oxidation. The NaClO‐based oxidation processes described by Liu and co‐workers were assessed with a LCA which showed that their processes are low‐carbon and cost‐effective technologies and therefore more sustainable than conventional processes.^[43]^

Mediated electrochemical processes can enable the circumvention of extensive chemical use, and therefore potentially make LFP recycling more sustainable and/or economically feasible. The LCA and TEA studies in some of the mentioned studies also support claims in this direction. The redox flow desalination coupled to LFP recycling process by Shan and co‐workers was assessed with the EverBatt model.^[65]^ This analysis showed that their process would result in significantly lower greenhouse gas emission and energy consumption, and is in contrast to pyro‐ and hydrometallurgical processes more profitable (a postulated revenue of approximately $1.87 per kg spent LFP cell).^[35]^ The redox flow battery system coupled with LFP recycling and selective lithium extraction from brine described by Li and co‐workers was evaluated by a TEA. Their process was shown to have a high lithium extraction rate at a low energy consumption with low process costs and is therefore more profitable compared to other lithium extraction technologies.^[53]^ Utilizing such a “dual‐mode” electrochemical process, they were able to boost the profitability and reported that this process costs less ($2.9 per extracted kg lithium) compared to extraction solely from a liquid or solid phase ($4.2 and $5.5 per extracted kg lithium, respectively). The process disclosed by Zhu and co‐workers using PMA as RM was compared to conventional pyro‐, hydro‐, and direct recycling methods by technoeconomic and environmental analyses showing that their process is both more sustainable and profitable.^[55]^ The authors analyzed their PMA mediated process with the EverBatt 2020 model, which showed a significant decrease in total energy consumption (2.3 MJ/kg LFP cell for the PMA process) compared to 12.1 and 19.6 MJ/kg LFP cell for the pyrometallurgical and hydrometallurgical routes, respectively. In addition, an economic analysis revealed that the profit of the redox‐mediated process was the highest at $2.67/kg sLFP, compared to pyro, hydro and direct recycling ($−0.45, $1.71, $2.51 respectively). Zhu and co‐workers attributed this increase in profit to faster reaction kinetics, less reagent consumption, and high revenues of the products.

It is important to keep in mind that the TEA evaluation of low TRL processes can only be considered indicative and often changes as the process is upscaled. Furthermore, the analyses described above are based on relatively pure LFP cathode material, which is challenging to obtain by manual dismantling at industrial scale. These examples have not fully considered utilizing black mass from processed LFP batteries as input. Black mass contains impurities like Cu and Al, and also consists of graphite and fluoride, making a true comparison with conventional methods challenging. Careful selection of an appropriate RM could enable high selectivity, but more research towards this is required to enable similar flexibility of an electrochemical leaching method and following separation/purification steps compared to conventional hydrometallurgical processing.

Similar to indirect electrochemical leaching, direct electrochemical leaching can also enable a more sustainable and economical way to recycle LFP batteries. Implementing this method on a large scale remains challenging due to the necessity of contact between the material and the electrode surface for effective electron transfer. However, alternative approaches, such as slurry electrolysis or innovative battery designs, may offer promising solutions for realizing this technique in the future. Apart from the technological challenges, the economic feasibility of direct electrochemical leaching was demonstrated by a number of studies. The process reported by Zhao and co‐workers was compared to conventional pyrometallurgical and hydrometallurgical processes using the Everbatt model.^[65]^ It was found that their method outperforms these processes in safety, revenue, energy efficiency and result in lower emissions.^[57]^ They reported that their direct electrochemical approach only requires 54 % of the total energy consumption compared to a conventional hydrometallurgical process. This leads to much lowered GHG emissions (approx. 1 kg/kg sLFP cell, 41 % reduction), compared to a conventional hydrometallurgical process. The authors also note that conventional pyro‐ and hydrometallurgical processes require expensive equipment for high temperatures and corrosive reagents, which increases their operational costs. However, they also rightfully acknowledge that these direct electrochemical recovery methods are still at lab‐scale development, which does not provide any accurate economic judgement. A preliminary economic and technical evaluation of the process described by Yang and co‐workers supported that their re‐lithiation‐de‐lithiation process is more sustainable and profitable compared to current pyro‐ and hydrometallurgical processes.^[59]^ The authors performed an economic assessment of their sLFP recycling process. This analysis shows a potential profit of $6923 per tonne processed sLFP. They also state that due to no requirement for external lithium source, no waste, and reduced energy consumption their proposed process also provides environmental benefits. The two slurry electrolysis methods described by Li and co‐workers also include an economic analysis which showed that their methods have low reagent and energy costs, making their processes economically feasible.[^61^, ^62^] They report that their approaches could make a potential revenues of $2846 per tonne sLFP,^[61]^ or $1567 per tonne sLFP,^[62]^ depending on the technology.

Although these reported numbers for environmental benefits, profits and revenues are not directly comparable, they do show a trend: replacing the use of chemicals for electrons (thus shifting from classic chemistry to electrochemistry) shows a lot of potential to both improve the economic aspects of a process, as well as the environmental aspects. What typically is considered as a tradeoff (economics versus the environmental benefits) appears in this innovative direction to be a win‐win.

## Conclusions and Outlook

LFP batteries, that are safer, cheaper and more stable compared to other LIB chemistries, are vital for the energy transition. However, large waste streams resulting from the increased demand for LFP batteries need to be addressed properly in the future. This offers the opportunity to recover critical raw materials, to support a circular economy and prevent release of toxic compounds into the environment. Therefore, it is essential to develop LFP battery recycling processes that are both sustainable and economically viable.

State‐of‐the‐art hydrometallurgical recycling methods involve the use of various acids and H₂O₂ as an oxidant. These offer a more feasible alternative to pyrometallurgical recycling but still require large amounts of chemicals and energy and result in waste streams. Alternative leaching methods using inexpensive oxidants show improvements, of which a few described processes have reduced environmental impact compared to the state‐of‐the‐art processes. Nonetheless, these processes still require the use of stoichiometric or excess amount of chemicals. In contrast, electrochemical methods present a more sustainable and profitable approach by minimizing chemical usage and enabling the regeneration of reagents. In this Concept, examples of such electrochemically driven leaching processes were discussed. As demonstrated by the examples from literature, such processes can enable high lithium recovery yields and have a lower environmental impact and operation costs, compared to conventional recycling methods.

Future research is expected to focus on optimizing electrochemical strategies for both leaching lithium, as well as recovering lithium compounds, and to explore new electrochemical opportunities. Electrochemical methods can be designed in a flexible way, and can be coupled to other useful electrochemical processes, enhancing overall efficiency and profitability. For example, electrochemical LFP recycling can be coupled with processes like green hydrogen production, brine desalination or can be combined with a reductive (co−)leaching process. To further boost the economic feasibility and sustainability of electrochemical LFP recycling, future research could explore on extending the incorporation with other processes such as; CO_2_ reduction, electrowinning from other recycling processes, or organic electrosynthesis. In addition, improvement of direct and indirect electrochemical recycling methods that eliminate the consumption of reagents or the production of waste should be further researched. This can lead to a more sustainable and economically viable solution for LFP battery recycling.

## Conflict of Interests

The authors declare no conflict of interest.

## Biographical Information

*Dr. Devin H.A. Boom obtained his PhD in 2018 at the University of Amsterdam in the field of main‐group and organometallic chemistry. Currently, he serves as a lead scientist at The Netherlands Organization for Applied Scientific Research (TNO). His research focuses on the development of sustainable recycling processes for the recovery of critical and strategic materials from waste streams. Specifically, his interests lie in the electrification of chemical recycling processes in order to regenerate reagents and increase the sustainability of the overall processes*.

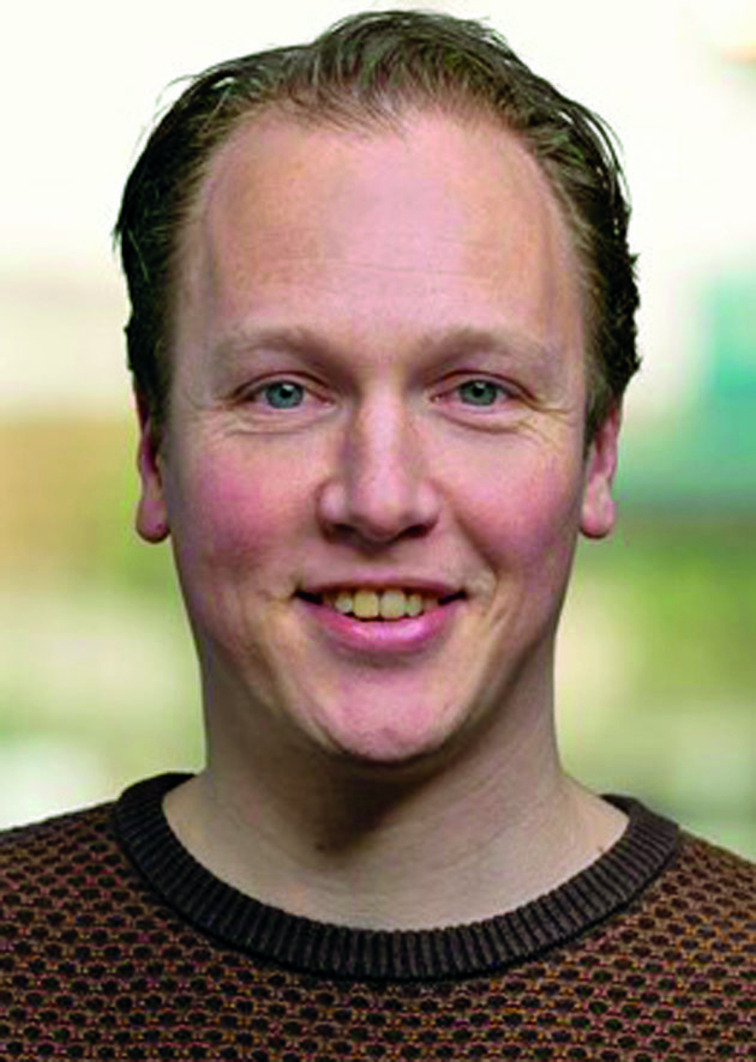

## Biographical Information

*Dr. Eda Yilmaz graduated from Bilkent University Chemistry Department in 2006. She studied characteristics of gold nanoparticles in polymer matrices using x‐ray photoelectron spectroscopy during her Ph.D. in Chemistry, which she received in 2011. Then she started to investigate lithium‐oxygen batteries and advanced materials for next generation lithium‐ion batteries, first as a postdoctoral researcher in RIKEN Institute in Japan then as a principle investigator in National Nanotechnology Research Center (UNAM) in Turkey. She currently works as a scientist in TNO at Circular Electronics team, with the aim of developing sustainable recycling processes for Li‐ion batteries and electronics waste*.

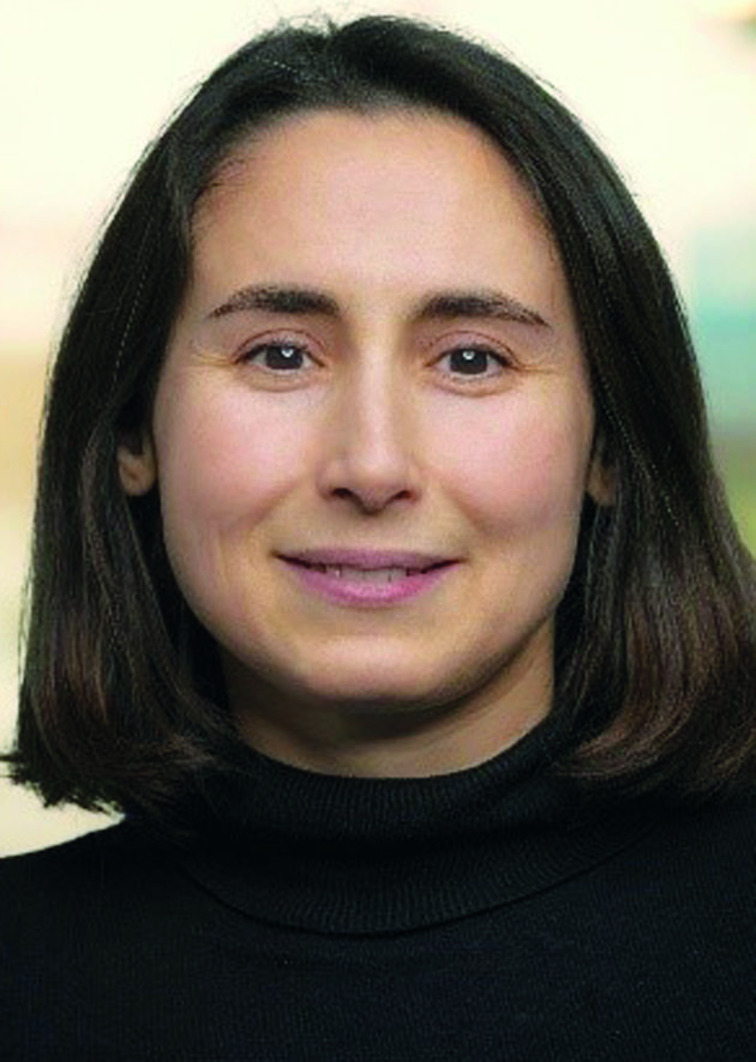

## Biographical Information

*Dr. Cody van Beek has obtained his Master′s degree at Utrecht University in the field of Nanomaterials Science in 2019. Afterwards, he continued with his PhD work in synthetic organometallic chemistry and multimetallic catalysis in the Organic Chemistry & Catalysis group at Utrecht University and graduated in 2024. In 2023 he joined The Netherlands Organization for Applied Scientific Research (TNO) as scientist. There, he works on the development of sustainable recycling technologies for recovery of critical materials from the urban mine by utilization of efficient electrochemical methods*.